# Haploinsufficiency of the Myc regulator Mtbp extends survival and delays tumor development in aging mice

**DOI:** 10.18632/aging.101092

**Published:** 2016-10-30

**Authors:** Brian C. Grieb, Kelli Boyd, Ramkrishna Mitra, Christine M. Eischen

**Affiliations:** ^1^ Department of Pathology, Microbiology and Immunology, Vanderbilt University Medical Center, Nashville, TN 37212, USA; ^2^ Department of Cancer Biology, Sidney Kimmel Cancer Center, Thomas Jefferson University, Philadelphia, PA 19107, USA

**Keywords:** Mtbp, Myc, aging, longevity, lymphoma

## Abstract

Alterations of specific genes can modulate aging. Myc, a transcription factor that regulates the expression of many genes involved in critical cellular functions was shown to have a role in controlling longevity. Decreased expression of Myc inhibited many of the deleterious effects of aging and increased lifespan in mice. Without altering Myc expression, reduced levels of Mtbp, a recently identified regulator of Myc, limit Myc transcriptional activity and proliferation, while increased levels promote Myc-mediated effects. To determine the contribution of Mtbp to the effects of Myc on aging, we studied a large cohort of *Mtbp* heterozygous mice and littermate matched wild-type controls. Mtbp haploinsufficiency significantly increased longevity and maximal survival in mice. Reduced levels of Mtbp did not alter locomotor activity, litter size, or body size, but *Mtbp* heterozygous mice did exhibit elevated markers of metabolism, particularly in the liver. *Mtbp*^+/−^ mice also had a significant delay in spontaneous cancer development, which was most prominent in the hematopoietic system, and an altered tumor spectrum compared to *Mtbp^+/+^* mice. Therefore, the data suggest Mtbp is a regulator of longevity in mice that mimics some, but not all, of the properties of Myc in aging.

## INTRODUCTION

Aging is a complex biological process controlled by both environmental and genetic factors [1]; however, twin studies suggest 20-30% of lifespan variation is genetic [2, 3]. Altering the activity or expression of specific genes significantly impacts lifespan in animal models [4]. For example, increased expression of the protein deacetylase Sirt1 is known to slow the effects of aging and increase lifespan [5]. In contrast, reduced levels of the oncogenic transcription factor c-Myc (Myc), due to heterozygosity, was recently reported to significantly increase longevity in mice [6–8].

Myc is estimated to transcriptionally regulate 10-15% of the genome [9, 10]. While Myc has been implicated in processes such as stem cell maintenance, differentia-tion, and apoptosis, Myc transcriptional activity is closely linked to cell-cycle progression and the vast metabolic machinery required for cellular proliferation [6, 7, 11]. Notably, Myc regulates mitochondrial biogenesis through expression of genes such as *Pgc1*ɑ and *Pgc1*β (peroxisome proliferation activated receptor gamma coactivator 1-alpa and beta), providing sufficient mitochondria to maintain increased cellular metabolism [12]. Myc increases overall cellular energy flux by upregulating glycolysis and glutaminolysis through transcriptional activations of target genes like hexokinase 2 (*Hk2*) and glutaminase (Gls; [13–16]). The energy generated from these metabolic pathways is then utilized by downstream pathways regulated by Myc to generate critical macromolecules. For example, Myc regulates urea cycling and pyrimidine synthesis, via transcriptional regulation of ornithine decarboxylase (*Odc*) and carbamoyl-phosphate synthetase 2/aspartate transcarbamylase/dihydroorotase (*Cad*), respectively [17, 18]. Myc also increases overall protein synthesis [19], a known modulator of longevity [20], through regulation of genes like nucleolin (*Ncl*) that control ribosomal assembly [21].

Based on the broad control Myc exerts over cellular processes relevant to aging and the recent publication directly linking Myc to longevity [8], proteins that regulate Myc represent potential modulators of the aging process. We recently reported that Mtbp is a Myc transcriptional co-factor [22]. In mice, *Mtbp* heterozygosity resulted in reduced Mtbp protein expression without altering Myc levels, and this inhibited Myc-mediated transcriptional activation of target genes, proliferation, and B cell lymphoma development [23]. Knockdown of *Mtbp* expression delayed cell cycle progression through S and G2/M phases of the cell cycle [24, 25]. In contrast, elevated Mtbp expression increased the number of cells in S-phase and enhanced Myc-mediated transcription and tumor development [22]. These data indicate Mtbp is a positive regulator of Myc transcriptional activity and downstream biological functions. Thus, we tested whether reduced Mtbp expression would alter aging in similar ways to decreased Myc expression [8].

## RESULTS

### *Mtbp^+/−^* mice have increased longevity

**Figure 1:**
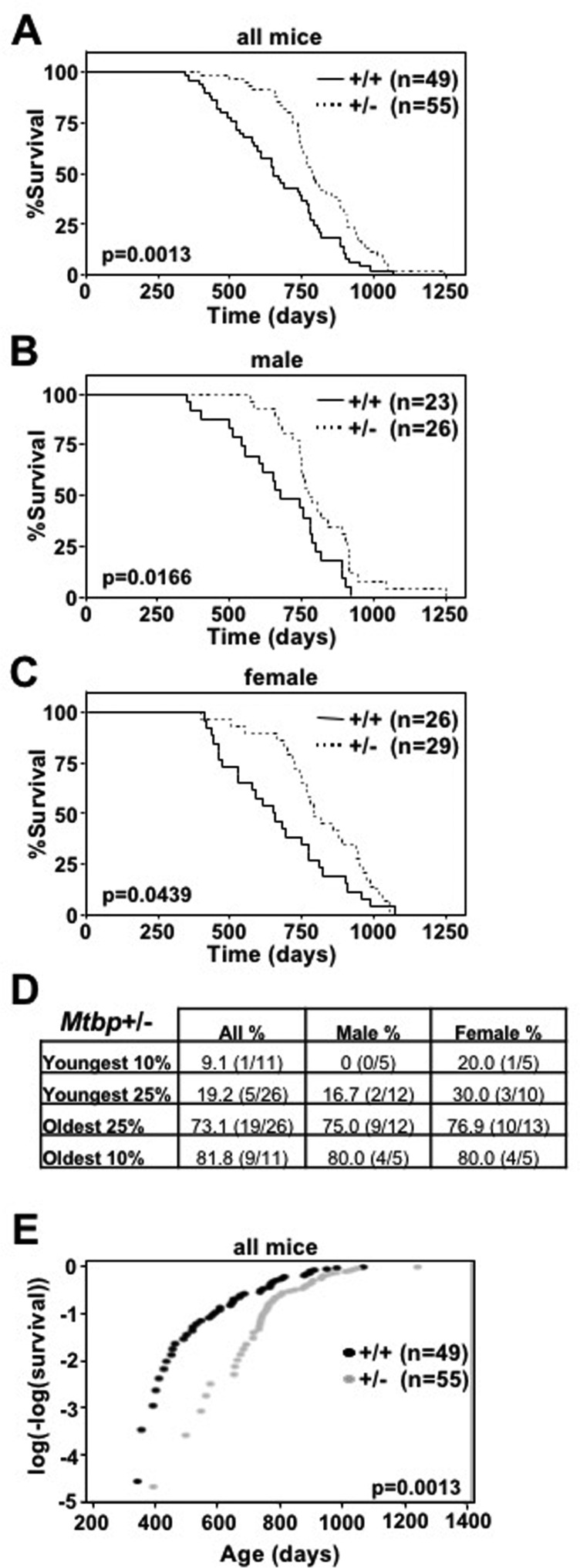
*Mtbp* heterozygosity increases longevity (**A**-**C**) Kaplan-Meier survival curves of *Mtbp*^+/+^ (+/+) and *Mtbp*^+/−^ (+/−) mice with the number of mice in each group denoted by n. p value determine by log-rank tests. (**D**) All, male, and female *Mtbp*^+/−^ mice in the indicated decile or quartile of the mouse cohort. (**E**) Instantaneous death rate plotted; log-rank *p*=0.0013, Chi-sq=10.27, df=1; number of mice in each group denoted by n.

Since *Myc*^+/−^ mice have increased longevity [8] and we have shown that Mtbp is a positive regulator of Myc [22, 23], we investigated the contribution of Mtbp to longevity using a cohort of littermate-matched *Mtbp*^+/+^ and *Mtbp*^+/−^ mice. *Mtbp* heterozygous mice had increased longevity compared to wild-type controls, exhibiting a median survival of 785 days compared to 654 days (p=0.0013; Figure 1A, Supplemental Figure S1), a 20% increase. This significant difference in lifespan was represented in both male and female populations (Figure 1B and 1C). *Mtbp* heterozygous males had a median survival of 774 days, compared to 672 days for wild-type control males (p=0.0166, Supplemental Figure S1), a 15.2% increase. *Mtbp*^+/−^ females had a median survival of 790 days, compared to 650.5 days for *Mtbp*^+/+^ females (p=0.0439), a 21.4% increase (Supplemental Figure S1).

In addition to median lifespan, *Mtbp* heterozygosity also increased maximum lifespan. Specifically, *Mtbp^+/−^* mice were overrepresented in the longest living decile and quartile of mice with 9 of 11 (81.8%) and 19 of 26 (73.1%) of the mice, respectively (Figure 1D). The trend was not affected by gender, as the longest living decile and quartile of mice were 4 of 5 (80.0%) and 9 of 12 (75.0%) heterozygous males, respectively, and 4 of 5 (80.0%) and 10 of 13 (76.9%) heterozygous females, respectively. In contrast, *Mtbp* wild-type mice (all mice and both male and female) were disproportionally represented in the shortest lived decile and quartile of mice 90.9% and 80.8%, respectively (Figure 1D). This was also reflected in the observation that *Mtbp*^+/−^ mice have a significantly decreased instantaneous death rate compared to *Mtbp*^+/+^ littermate-matched controls (log-rank p=0.0013, Chi-sq=10.27, df=1; Figure 1E). Both *Mtbp* heterozygous males and females have a significantly reduced instantaneous death rate (log-rank p=0.0166, Chi-sq=5.74, df=1 and log-rank p=0.0439, Chi-sq=4.06, df=1, respectively; Supplemental Figure S2). Therefore, an Mtbp haploinsufficiency confers increased median and maximum survival.

### Delay in tumorigenesis and a change of tumor spectrum in *Mtbp* heterozygous mice

**Figure 2:**
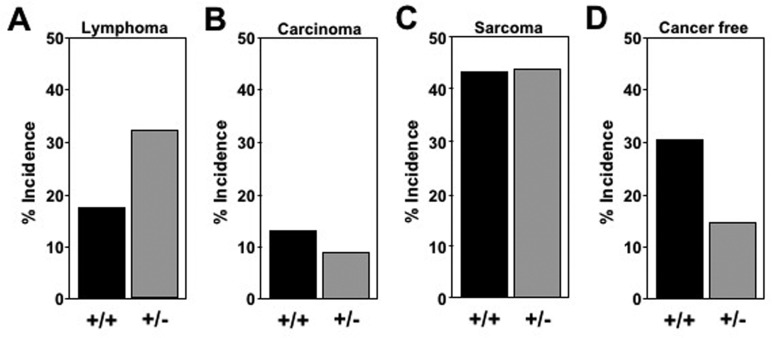
Altered tumor spectrum in *Mtbp^+/−^* mice Pathological/histological evaluation of tissues at time of death of *Mtbp^+/−^* n=23 and *Mtbp^+/−^* n=34 littermates analyzed. The percentage of mice with each diagnosis plotted (**A**-**D**).

As is commonly seen in C56Bl/6 mice [26, 27], gross and histopathological tissue analysis at time of death of representative mice demonstrated the majority had cancer (17 of 23 *Mtbp*^+/+^ mice and 29 of 34 *Mtbp*^+/−^ mice). Notably, 32.4% (11 of 34) of *Mtbp*^+/−^ mice had lymphoma, which was twice the incidence of lymphoma in *Mtbp*^+/+^ mice (17.4%, 4 of 23; Figure 2A). The lymphomas were detected at an average age of 840 days in heterozygotes, compared to 682.3 days in wild-type controls, a significant delay (p=0.0320, Supplemental Figure S1). Similarly, *Mtbp*^+/−^ mice developed carcinomas later in life at 848 days (3 of 34, 8.8%) compared to 694 days for *Mtbp*^+/+^ mice (3 of 23, 13.0%) and the tissue distribution of the carcinomas differed between the two genotypes. Specifically, two carcinomas that developed in the wild-type mice were hepatocellular carcinoma and one was a carcinoma of the small intestine, whereas two of the *Mtbp* hetero-zygous mice developed aural squamous cell carcinoma and one had pulmonary adenocarcinoma. The difference in age of carcinoma development and the type of carcinoma that emerged between the two genotypes was not statistically significant likely due to the small number of carcinomas that developed (p=0.1412; Supplemental Figure S1, Figure 2B). In contrast, *Mtbp*^+/+^ and *Mtbp*^+/−^ mice had a similar frequency and age of onset of sarcoma. Approximately half of the cancers that developed in both genotypes of mice were sarcoma [10 of 23 (43.5%) for *Mtbp*^+/+^ and 15 of 34 (44.1%) for *Mtbp*^+/−^; Figure 2C], occurring at a mean age of 806 days for *Mtbp*^+/+^ and 743 days for *Mtbp*^+/−^ mice (p = 0.1547, Supplemental Figure S1). The vast majority of the sarcomas in both the wild-type and *Mtbp* heterozygous mice were histiocytic sarcomas. One *Mtbp* wild-type mouse developed both a sarcoma and a carcinoma, and all *Mtbp*^+/−^ mice that were diagnosed with a malignancy had only one tumor type. Although, twice the proportion of *Mtbp* wild-type control mice were cancer free at time of death (7 of 23, 30.4%) compared to *Mtbp* heterozygous mice (5 of 34, 14.7%; Figure 2D), the *Mtbp*^+/−^ mice lived an average of 836.4 days compared to 640.3 days for wild-type controls (Supplemental Figure S1). This difference in *Mtbp*^+/−^ mice represents a significant delay in mortality among cancer free mice (p=0.03340). These data collectively indicate a decrease in Mtbp expression alters the tumor spectrum and age of onset as mice age, as well as extends overall survival independent of cancer development.

### *Mtbp^+/+^* and *Mtbp^+/−^* mice move, reproduce, and develop similarly

**Figure 3:**
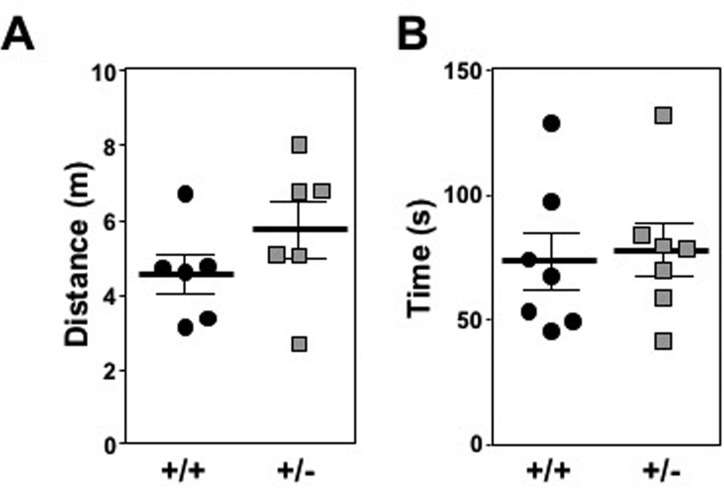
*Mtbp* heterozygosity does not significantly alter locomotor activity in old mice (**A**) *Mtbp*^+/+^ (+/+; circle) and *Mtbp*^+/−^ (+/−; square) mice were placed in an open field cage and the total distance traveled in one hour was recorded using a laser grid and averaged for two consecutive days (p=0.1142). (**B**) After two days of training, the time mice spent on an accelerating rota-rod was recorded and averaged from three consecutive trials separated by 10 minutes of rest (p=0.3923). P values calculated with student's t-tests and error bars are SEM.

Long-lived mouse models will often retain elevated motor function compared to controls, particularly as they age. To determine if *Mtbp* heterozygosity improved locomotor activity, open field testing was performed for 1 hour on two days with a cohort of old (1.5 year) littermate matched mice. Although there was a trend for *Mtbp* heterozygotes to travel a greater distance (5737.7 cm) compared to wild-type controls (4551.0 cm), this difference did not reach statistical significance (p=0.1142; Figure 3A). When locomotor function was actively challenged using a rota-rod endurance test, the *Mtbp*^+/−^ mice (78.0 seconds) performed similarly to Mtbp+/+ mice (73.6 seconds) after training (Figure 3B; p=0.3923). Analogous results were also obtained with younger mice (Supplemental Figure S3).

**Figure 4:**
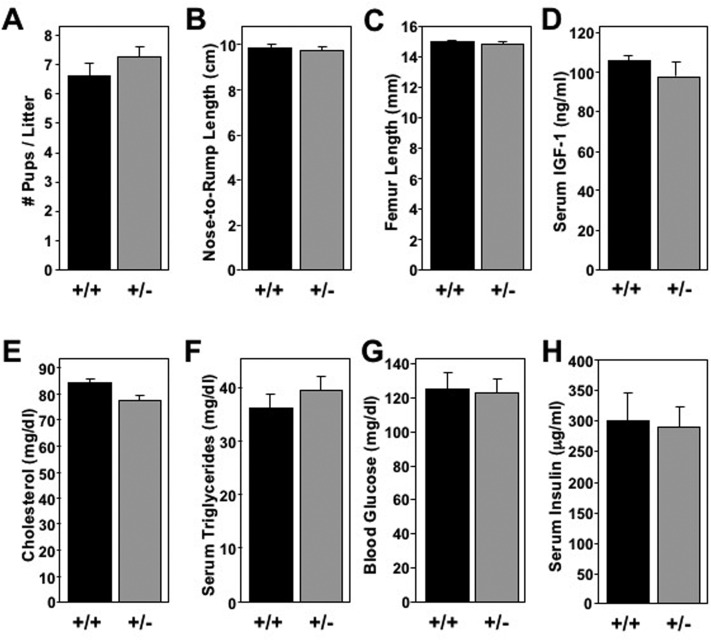
Long-lived *Mtbp* heterozygous mice exhibit normal systemic physiology (**A**) The mean number of pups birthed from *Mtbp*^+/+^ x *Mtbp*^+/−^ crosses by each female *Mtbp*^+/+^ (+/+; n=20 females; 328 total pups; black) and *Mtbp*^+/−^ (+/−; n=25 females; 569 total pups; gray) mouse was recorded and averaged (p=0.2247). (**B**-**H**) Healthy long-lived (29 months) +/+ (n=5) and +/− (n=8) male mice were starved for 5 hours. (**B**) The nose-to-rump length was recorded and averaged (p=0.9999). (**C**) After sacrifice, the femurs were isolated and their length measured with electronic calipers and averaged (p=0.7160; n=7 for +/− group due to loss of one femur from bilateral fracture from collection). (**D**-**H**) Blood was collected and serum isolated. Circulating levels of (**D**) IGF-1 (p=0.4175), (**E**) cholesterol (p=0.3572), (**F**) triglycerides (p=0.4037), (**G**) blood glucose (p=0.7116) and **(H)** insulin (p=0.6963) were measured. P values calculated with student's t-tests, and error bars are SEM.

In nature, many animal species with increased longevity have reduced reproductive capacity to limit overpopulation. This trend has been reported in some long-lived mouse models [28]. Thus, we compared the reproductive efficiency of *Mtbp*^+/+^ and *Mtbp*^+/−^ female mice. Only the number of pups per birth from crosses between *Mtbp*^+/+^ and *Mtbp*^+/−^ mice were quantified, as deletion of *Mtbp* is embryonic lethal and would artificially lower the number of pups birthed [29]. This examination did not reveal a significant difference in the average number of pups per litter birthed by *Mtbp*^+/+^ (6.6) and *Mtbp*^+/−^ (7.3) females (p=0.2247; Figure 4A).

Some long-lived mouse models reported to have reduced growth, resulting in smaller body size [30]. We detected no size differences in mature *Mtbp*^+/−^ mice. Specifically, *Mtbp*^+/+^ and *Mtbp*^+/−^ mice had similar nose-to-rump lengths of 9.89 cm and 9.74 cm, respectively (p=0.9999; Figure 4B) as well as femur lengths of 15.0 mm and 14.8 mm, respectively (p=0.7160; Figure 4C) at the time of sacrifice. Given this observation, it was not surprising that analysis of serum isolated and frozen at time of sacrifice did not show a statistically significant difference in the level of circulating insulin- like growth factor-1 (IGF-1; p=0.4175; Figure 4D), a major growth-promot-ing factor [31]. Therefore, an Mtbp haploinsufficiency did not impact locomotion, birth rates, or bone size.

### Long-lived *Mtbp^+/−^* mice exhibit signs of increased cellular metabolism

Many long-lived mouse models have changes in metabolism detectable at a systemic level. To determine if Mtbp expression modulates levels of circulating metabolic markers, serum was isolated and frozen immediately after sacrifice of long-lived mice starved for 5 hours. The analysis revealed *Mtbp*^+/+^ and *Mtbp*^+/−^ mice had similar levels of serum cholesterol (p=0.3572; Figure 4E) and triglycerides (p=0.4037; Figure 4F). Moreover, the circulating level of glucose (p=0.7116; Figure 4G) and insulin (p=0.6963; Figure 4H) were also similar, reflecting no major changes in physiologic glucose regulation.

**Figure 5:**
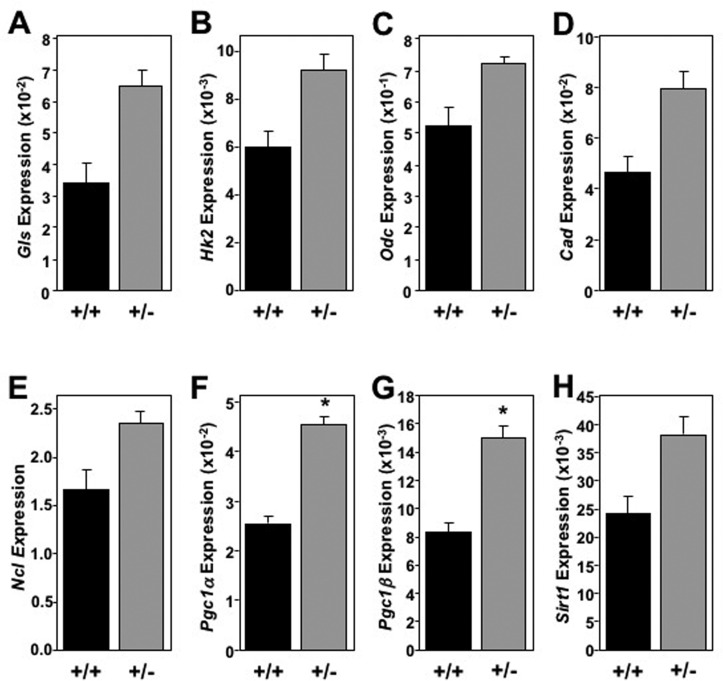
Old *Mtbp* heterozygous mouse livers exhibit global elevated metabolic markers (**A**-**H**) Healthy *Mtbp*^+/+^ (+/+; n=5; black) and *Mtbp*^+/−^ (+/−; n=9; gray) mice at 29 months were starved for 5 hours, sacrificed, and livers frozen with Wallenburg clamp. qRT-PCR for **(A)** glutaminase (*Gls*, p=0.1147), (**B**) hexokinase 2 (*Hk2*, p=0.1401), (**C**) ornithine decarboxylase (*Odc*, p=0.0736), (**D**) carbamoyl-phosphate synthetase 2/aspartate transcarbamylase/dihydroorotase, (*Cad*, p=0.1393), (**E**) nucleolin (*Ncl,* p=0.1412), (**F**) peroxisome proliferation activated receptor gamma coactivator 1-alpha (*Pgc1*ɑ, *p=0.0106), (**G**) *Pgc1*-beta (*Pgc1*β, *p=0.0499), and (**H**) sirtuin-1 (*Sirt1*, p=0.1529) was performed. Values are relative to *β-actin* levels. P values calculated using student's t-tests and error bars are SEM.

Although systemic changes in cholesterol, triglyceride, and glucose metabolism were not observed, we examined markers of cellular metabolism in tissues of long-lived mice to determine if decreased Mtbp expression modulates metabolism at a molecular level. Using flash frozen tissue at the time of sacrifice, mRNA was isolated from the liver, skeletal muscle (gastrocnemius) and brown fat pad of long-lived mice. In the liver, *Mtbp*^+/−^ mice exhibited a global trend toward, and at times significantly, increased expression of metabolic markers (Figure 5), similar to previous reports for *Myc*^+/−^ mice [8]. *Mtbp* heterozygosity resulted in a trend toward increased expression of basic metabolic genes such as *Gls*, *Hk2*, *Ncl*, *Cad*, and *Odc* that control cellular energy flux, protein translation, and macromolecule synthesis (Figure 5A-E). Most notably, the livers of *Mtbp*^+/−^ mice showed a nearly 2-fold and statistically significant increase in the expression of *Pcg1*ɑ (p=0.0106; Figure 5F) and *Pcg1*β (p = 0.0499; Figure 5G) compared to wild-type controls. This increase suggests elevated mitochondrial biogenesis and function in long-lived *Mtbp* heterozygous mice. These increased levels of metabolic markers in *Mtbp*^+/−^ livers also coincided with a nearly 60% increase in the expression of *Sirt*1 (p=0.1529; Figure 5H), a well-known anti-aging gene linked to caloric restriction [32].

**Figure 6:**
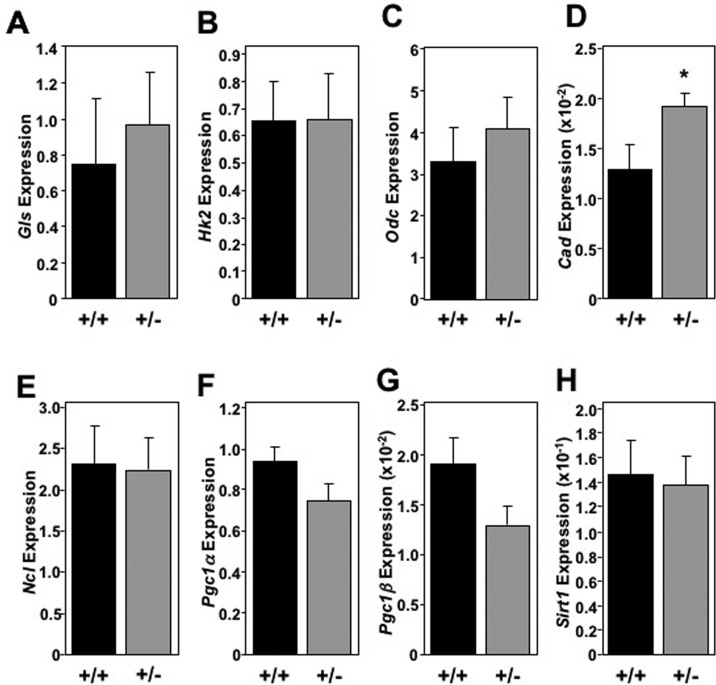
Skeletal muscle in old *Mtbp*^+/−^ mice lack global metabolic marker increase (**A**-**H**) Healthy *Mtbp*^+/+^ (+/+; n=5; black) and *Mtbp*^+/−^ (+/−; n=8; gray) mice at 29 months were starved for 5 hours, sacrificed, and gastrocnemius muscle frozen with Wallenburg clamp. qRT-PCR for **(A)** glutaminase (*Gls*, p=0.5591; +/− n=7 due to RNA loss), (**B**) hexokinase 2 (*Hk2*, p=0. 9792), (**C**) ornithine decarboxylase (*Odc*, p=0.5115), (**D**) carbamoyl-phosphate synthetase 2/aspartate transcarbamylase/dihydroorotase, (*Cad*, *p=0.0202), (**E**) nucleolin (*Ncl,* p=0.9116, +/− n=7 due to insufficient RNA), (**F**) peroxisome proliferation activated receptor gamma coactivator 1-alpha (*Pgc1*ɑ, p=0.0736), (**G**) *Pgc1*-beta (*Pgc1*β, p=0.0710), and (**H**) sirtuin-1 (*Sirt1*, p=0.8417) was performed. Values are relative to *β-actin* levels. P values calculated using student's t-tests and error bars are SEM.

**Figure 7:**
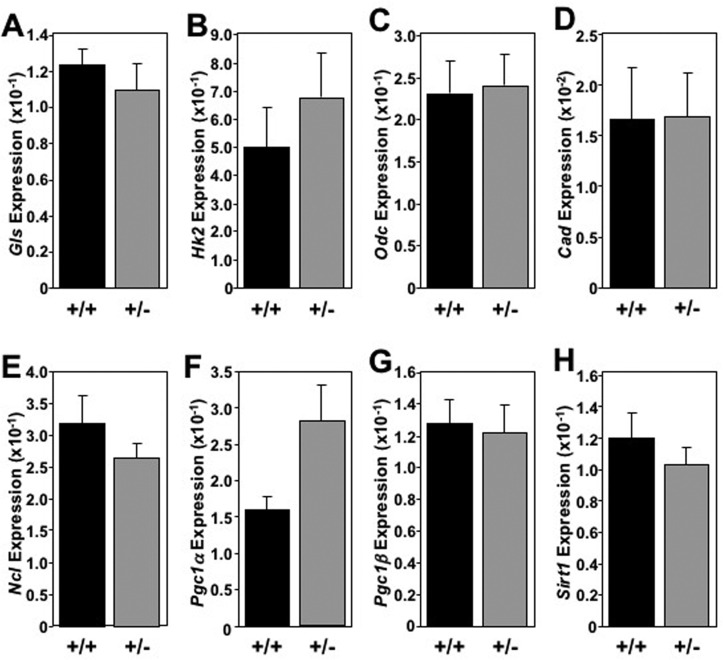
Absence of global elevation of metabolic markers in brown fat in old *Mtbp* heterozygous mice **(A**-**H**) Healthy *Mtbp*^+/+^ (+/+; n=5; black) and *Mtbp*^+/−^ (+/−; n=8; gray) mice at 29 months were starved for 5 hours, sacrificed, and brown fat frozen. qRT-PCR for (**A**) glutaminase (*Gls*, p=0.4982), (**B**) hexokinase 2 (*Hk2*, p=0.4555), (**C**) ornithine decarboxylase (*Odc*, p=0.8677), (**D**) carbamoyl-phosphate synthetase 2/aspartate transcarbamylase/dihydroorotase, (*Cad*, p=0.9700), (**E**) nucleolin (*Ncl,* p=0.2668), (**F**) peroxisome proliferation activated receptor gamma coactivator 1-alpha (*Pgc1*ɑ, p=0.0531), (**G**) *Pgc1*-beta (*Pgc1*β, p=0.8492, +/+ n=4 due to insufficient RNA), and (**H**) sirtuin-1 (*Sirt1*, p=0.4146) was performed. Values are relative to *β-actin* levels. P values calculated using student's t-tests and error bars are SEM.

The global increase in metabolic markers observed in the livers of *Mtbp* heterozygous mice was largely not recapitulated in skeletal muscle (Figure 6) and brown fat (Figure 7). In skeletal muscle for example, there was only a statistically significant increase in the level of *Cad* in *Mtbp*^+/−^ mice (p=0.0202), but analogous levels of *Gls*, *Hk2*, *Odc*, and *Ncl* compared to *Mtbp*^+/+^ mice (Figure 6A-E). There was a trend toward significantly decreased expression of *Pcg1*ɑ (p=0.0736; Figure 6F) and *Pcg1*β (p=0.0710; Figure 6G), but similar levels of *Sirt1* (Figure 6H) in *Mtbp* heterozygous skeletal muscle. In brown fat, levels of *Gls*, *Hk2*, *Odc*, *Cad*, and *Ncl* were similar between wild-type and heterozygous *Mtbp* mice (Figure 7A-E). However, the brown fat of *Mtbp*^+/−^ mice exhibited a trend toward increased expression of *Pcg1*ɑ (p=0.0531; Figure 7F), but equivalent levels of *Pcg1*β (p=0.8492; Figure 7G). The levels of Sirt1 were analogous between the two genotypes (Figure 7H). Therefore, the data show *Mtbp* heterozygosity alters markers of cellular metabolism in disparate tissues, although the effects are more pronounced in the liver.

## DISCUSSION

We reported that Mtbp is a positive regulator of Myc transcriptional activity, promoting Myc-mediated proliferation and malignant transformation [22, 23]. Yet, it was unclear if Mtbp expression contributed to Myc-modulation of aging that was recently reported [8]. Here, we determined *Mtbp* heterozygosity, like *Myc* heterozygosity, significantly increased the median and maximum lifespan of mice and delaying cancer development compared to wild-type littermate-matched controls. The increase was observed regardless of gender. These results indicate Mtbp has a significant role in aging.

While cancer was the cause of death in the majority of mice, reduced Mtbp expression was associated with an increased, but significantly delayed, incidence of lymphoma. We observed a similar delay in lymphoma development in Eμ-*myc* transgenic mice that were *Mtbp*^+/−^ [23]. Interestingly, *Myc*^+/−^ mice, also had an increase in the rate of lymphoma, although to a much less degree, along with significantly reduced progression of disease at time of death [8]. In addition to delayed lymphoma development, there was also a trend for *Mtbp*^+/−^ mice to have delayed carcinoma development. However, *Mtbp* heterozygous and wild-type mice had a similar age of onset of sarcoma development. Therefore, the development of specific cancer types appears to be more impacted than others by *Mtbp* heterozygosity. Specifically, the data suggest the hematopoietic compartment may be more sensitive to changes in Mtbp expression than other tissues. However, in human cancer, *MTBP* is amplified and/or overexpressed in a range of malignancies, including lymphomas, carcinomas, and sarcomas, suggesting it is oncogenic in multiple tissue types [22, 23, 33]. Future studies of Mtbp function comparing different normal and cancerous tissues should clarify those cell types in which altered Mtbp levels have a significant impact.

Our data that sarcomas present at a similar age suggest the overall survival difference between *Mtbp*^+/−^ and *Mtbp*^+/+^ mice does not appear to be due to an overall delay in cancer development. Additionally, fewer *Mtbp*^+/−^ mice were cancer-free at the time of death, a result not predicted had Mtbp only impacted longevity by decreasing and/or delaying cancer development. Notably, cancer-free *Mtbp*^+/−^ mice lived significantly longer than their cancer-free wild-type littermates, suggesting that in the absence of cancer, reduced Mtbp expression conferred a survival benefit. These data suggest that the delayed presentation of lymphoma may reflect increased vitality of the *Mtbp*^+/−^ mice, as lymphoma development occurred at younger ages in the wild-type cohort.

In addition to increased longevity and modulated cancer development, long-lived *Mtbp* heterozygous mice exhibited a global trend toward elevated cellular metabolism in the liver. While this may coincide with an overall increase in metabolism reported for *Myc*^+/−^ mice [8], specific ties to aging have also been described. For example, caloric restriction, well known to improve longevity, has been previously shown to increase hepatic *Gls* expression [34]. *Odc* expression is elevated in younger livers and been implicated in the capacity for hepatic repair and regeneration [35]. We also observed increased expression of *Pgc1*ɑ and *Pgc1*β, which regulate mitochondrial biogenesis and function. Their reduced expression with aging has been associated with many age-related pathologies [36]. Collectively, increased expression of these metabolic markers suggests retained vitality in the livers of old *Mtbp*^+/−^ mice, which coincides with the elevated expression of the well-known anti-aging gene *Sirt1* [5].

The increased expression of metabolic genes observed in aged *Mtbp*^+/−^ livers was largely sporadic or nearly absent in skeletal muscle and brown fat. Interestingly, the absence of global metabolic changes in the skeletal muscle and brown fat of *Mtbp*^+/−^ mice compared to the global increase observed in the liver also matches with a lack of elevated *Sirt*1 expression observed in these tissues. Moreover, the lack of increase in metabolic markers and the downward trend in *Pgc1*ɑ and *Pgc1*β expression in skeletal muscle of *Mtbp*^+/−^ mice support their similar performance in open field and rota-rod testing compared to wild-type controls. This is in contrast to *Myc*^+/−^ mice, which showed metabolic changes in skeletal muscle and improved rota-rod performance [8]. The reasons for the differential response to decreased Mtbp expression in the liver compared to skeletal muscle and adipose tissue is unclear at this time. However, a microarray analysis of liver, skeletal muscle, and adipose tissue in old *Myc*^+/+^ and *Myc*^+/−^ revealed the highest number of differentially expressed genes occurred in the liver [8]. Thus, future studies focused on the tissue-specific benefits of reduced Mtbp and/or Myc expression would be important.

Collectively, the data suggest Mtbp impacts longevity and cellular metabolism, particularly in the liver. These results are in line with a recent report on Myc [8] as well as our previous reports indicating Mtbp is a positive regulator of Myc transcriptional activity [22, 23]. However, the effect of *Myc* heterozygosity appears broader than the effects observed for *Mtbp* heterozygosity. For example, decreased Myc expression resulted in smaller body size, improved rota-rod performance, reduced circulating IGF-1, and lower serum cholesterol [8]. The precise reason for these differences is unclear at this time, although we have previously demonstrated Mtbp expression does not alter protein expression of Myc [22, 23], but did change the transcriptional activity of Myc. Part of the downstream effects of Myc are mediated through direct binding to or displacement of other factors at DNA, such as Miz1, NFY, C/EBPβ, SP1, and Foxo3A [7, 37, 38]. Myc also regulates transcriptional elongation through recruitment of P-TEFb [39, 40]. It is unknown how Mtbp expression impacts these functions of Myc or whether these functions of Myc change as animals age. Moreover, it is possible Mtbp may only orchestrate a sub-set of Myc's overall transcriptional activity and may have Myc-independent functions. Therefore, additional research is needed on the interaction between Mtbp and Myc, and Mtbp itself, to better understand the contribution of Mtbp to aging.

## METHODS

### Mice

*Mtbp^+/−^* [29] and littermate-matched *Mtbp^+/+^* C57Bl/6 mice of both genders were generated through interbreeding and were housed together. For survival analysis, mice were sacrificed after meeting humane end-of-life criteria. Necropsy with tissue collection was performed and tissues were evaluated by a board-certified veterinary pathologist (K.B.) in a blinded manner. For analysis of healthy aged mice (29 months-old), the mice were starved for 5 hours and crown-to-rump length was measured. Blood was collected and analyzed for blood glucose levels with Accu-chek test strips (Roche Diagnostics, Indianapolis, IN, USA; [41], centrifuged and plasma frozen for later analysis. Mice were sacrificed by cervical dislocation. Liver and gastrocnemius muscle were frozen with a Wallenburg clamp pre-cooled in liquid nitrogen as previously described [42]. Brown fat pads were collected and frozen. Tissues were kept at −80°C until analysis. Femurs were collected and measured with electronic calipers. Experiments were approved by the Vanderbilt Institutional Animal Care and Use Committee and followed all federal and state rules and regulations.

### Quantitative real-time PCR (RT-PCR)

Total RNA was isolated from frozen tissues, cDNA was generated, and qRT-PCR was performed as previously described [23]. Primer sequences are listed in supplemental material.

### Serum analysis

Analysis of serum was performed by the Mouse Metabolic Phenotyping Center in the Hormone Assay and Analytical Services and the Lipids and Lipoproteins Subcores at Vanderbilt University. Insulin levels were measured with a radioimmunoassay (SRI-13K, EMD Millipore, Billerica, MA, USA). IGF-1 levels were measured using a magnetic Luminex screening assay (LXSAMSM-01, R&D Systems, Minneapolis, MN, USA). Triglyceride and cholesterol levels were measured using Raichem reagents (R80035 and R84098, Cliniqa, San Marco, CA, USA).

### Locomotion analysis

Explorative locomotion or open field testing was performed using a 48 channel IR controller (ENV-520, Med Associates Inc., St. Albans, Vermont) on young (6 months-old) and aged (21 months-old) mice. On two different days, mice were placed in the open field, the total distance traveled was recorded for 1 hour and the average of the two measurements was reported. Additionally, on three consecutive days, mice were placed on a five-lane accelerating rota-rod (47600, Ugo Basile, Varese, Italy) for three sequential trials separated by 10 minutes of rest. The rota-rod accelerated from 4 to 40 rpm over 3 minutes with a cut-off time of 3 minutes. Time running on the rota-rod was determined by using pressure sensors to detect falls or by observing >3 consecutive rotations of the mouse around the rod. The average times from day 3 were recorded [43].

### Statistical evaluation

A log-rank test was used to calculate significance in Figure 1 and Supplemental Figure S1. Analysis for Figure 1E and Supplemental Figure S1 was performed with JMP statistical software (v12.2.0). Student's t-test was used to calculate significance for data in Figures 2–7 and Supplemental Figures S2 and S3.

## SUPPLEMENTARY MATERIALS FIGURES


