# Supplementary material for: Haploinsufficiency of the Myc regulator Mtbp extends survival and delays tumor development in aging mice
Source: Aging (Albany NY). 2016 Oct 30;8(10):2590–600. doi: 10.18632/aging.101092 (PMC5115908; doi:10.18632/aging.101092)
Supplement: Supplementary file 1 [file aging-08-2590-s001.pdf]

SUPPLEMENTAL MATERIAL

METHODS

Real Time PCR Primers

Cad-F – AACTGCGTAGGCTTCGACCATACA
Cad-R – AATCAATGCGGGTGAGCTCGTAGA
Gls [1]
Gls-F – TTCGCCCTCGGAGATCCTAC
Gls-R – CCAAGCTAGGTAACAGACCCT
Hk2 [2]
Hk2-F – TGATCGCCTGCTTATTCACGG
Hk2-F – AACCGCCTAGAAATCTCCAGA
Ncl-F – ACTGGAAAGACCAGCACTTGGAGT
Ncl-R – CCCTTTAGGTTTGCCATGTGGGTT
Odc-F – GCATGTGGGTGATTGGATGCTGTT
Odc-R – TTGCCACATTGGCCGTGACATTAC
Pcg1a-F – GGATGAATACCGCAAAGAGC
Pcg1a-R – GGTAGGTGATGAAACCATAGC
Pcg1b [3]
Pcg1b-F – TCCTGTAAAAGCCCGGAGTAT
Pcg1b-R – GCTCTGGTAGGGGCAGTGA
Sirt1 [4]
Sirt1-F – ACCTCCCAGACCCTCAAGC
Sirt1-R – TTCCTTCCTTATCTGACAAAGC

FIGURES

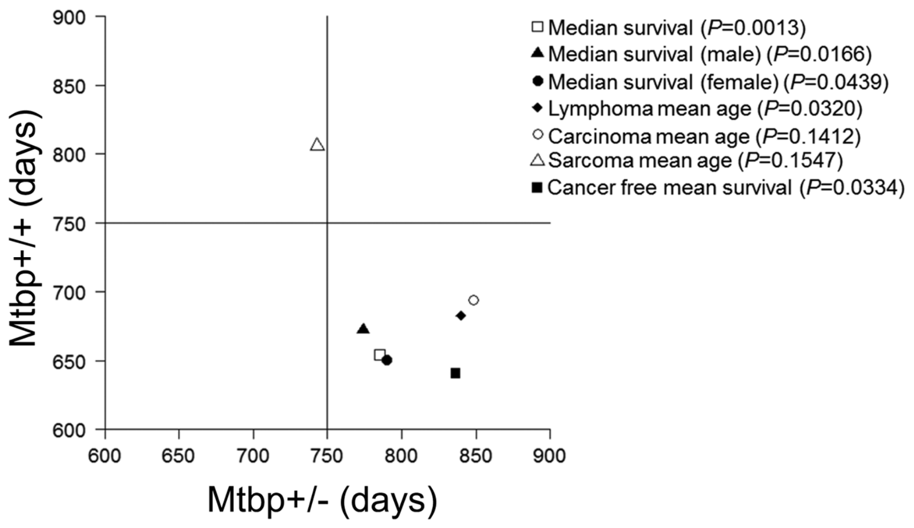

Figure S1. Indicators of increased longevity in *Mtbp+/-* mice. Ages of the events indicated in the key for *Mtbp+/-* mice compared to littermate matched *Mtbp+/+* mice plotted. P values determined by student's t-tests.

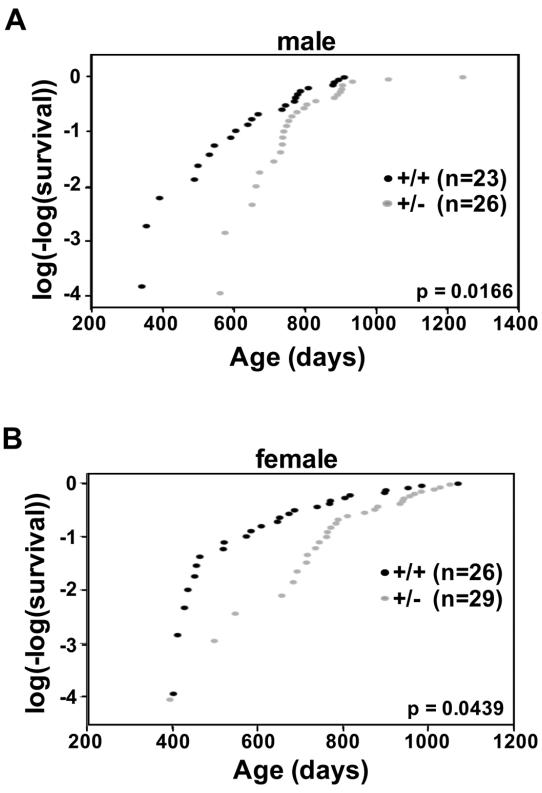

Figure S2. Male and female *Mtbp* heterozygous mice have a decreased instantaneous death rate. Instantaneous death rate plotted for males (**A**), log-rank *P* = 0.0166, Chi-sq=5.74, df=1) and females (**B**), log-rank *P* = 0.0439, Chi-sq=4.06, df=1). The number of mice in each group denoted by n.

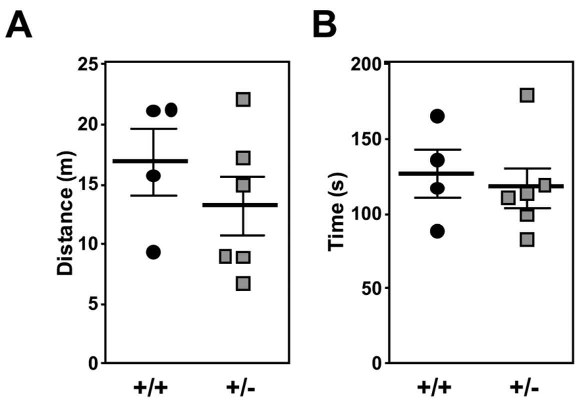

Figure S3. *Mtbp* heterozygosity does not significantly alter locomotor activity in young mice. (**A**) Six month-old *Mtbp*+/+ (+/+; circle) and *Mtbp*+/- (+/-; square) mice were placed in an open field cage and the total distance traveled in one hour was recorded using a laser grid and averaged for two consecutive days (p= 0.1772). (**B**) After two days of training, the time +/+ and +/- mice spent on an accelerating rota-rod recorded and averaged from three consecutive trials separated by 10 minutes of rest (p=0.3359). P values calculated with student's t-tests. Error bars represent standard error of the mean.

REFERENCES

1. Hettmer S, Schinzel AC, Tchessalova D, Schneider M, Parker CL, Bronson RT, Richards NG, Hahn WC and Wagers AJ. Functional genomic screening reveals asparagines dependence as a metabolic vulnerability in sarcoma. Elife. 2015; 4.

2. Shi LZ, Wang R, Huang G, Vogel P, Neale G, Green DR and Chi H. HIF1alphadependent glycolytic pathway orchestrates a metabolic checkpoint for the differentiation of TH17 and Treg cells. J Exp Med. 2011; 208: 1367-1376.

3. Walkey CJ and Spiegelman BM. A functional peroxisome proliferator-activated receptor-gamma ligand-binding domain is not required for adipogenesis. J Biol Chem. 2008; 283: 24290-24294.

4. Saini A, Al-Shanti N, Sharples AP and Stewart CE. Sirtuin 1 regulates skeletal myoblast survival and enhances differentiation in the presence of resveratrol. Exp Physiol. 2012; 97: 400-418.
